# Drug-induced autoimmune hepatitis due to atorvastatin: a complex clinical case and literature review

**DOI:** 10.1093/gastro/goag022

**Published:** 2026-03-07

**Authors:** Nancy H Serrano-Pérez, Jeanete S Rodríguez-Martínez, Sandra M Feria-Agudelo, Ana Lilia Morales-Leyte, Jacqueline Cordova-Gallardo

**Affiliations:** School of Medicine, National Autonomous University of Mexico, Escolar 411A, Copilco Universidad, Coyoacán, Mexico City, 04510, Mexico; Hepatology Department, Internal Medicine, Dr. Manuel Gea González General Hospital, Calzada de Tlalpan 4800, Seccion XVI, Tlalpan, Mexico City, 14080, Mexico; School of Medicine, National Autonomous University of Mexico, Escolar 411A, Copilco Universidad, Coyoacán, Mexico City, 04510, Mexico; Hepatology Department, Internal Medicine, Dr. Manuel Gea González General Hospital, Calzada de Tlalpan 4800, Seccion XVI, Tlalpan, Mexico City, 14080, Mexico; School of Medicine, National Autonomous University of Mexico, Escolar 411A, Copilco Universidad, Coyoacán, Mexico City, 04510, Mexico; Hepatology Department, Internal Medicine, Dr. Manuel Gea González General Hospital, Calzada de Tlalpan 4800, Seccion XVI, Tlalpan, Mexico City, 14080, Mexico; School of Medicine, National Autonomous University of Mexico, Escolar 411A, Copilco Universidad, Coyoacán, Mexico City, 04510, Mexico; Pathological Anatomy Department, Dr. Manuel Gea González General Hospital, Calzada de Tlalpan 4800, Colonia Sección XVI, Tlalpan, Mexico City, 14080, Mexico; School of Medicine, National Autonomous University of Mexico, Escolar 411A, Copilco Universidad, Coyoacán, Mexico City, 04510, Mexico; Hepatology Department, Internal Medicine, Dr. Manuel Gea González General Hospital, Calzada de Tlalpan 4800, Seccion XVI, Tlalpan, Mexico City, 14080, Mexico

**Keywords:** Drug-induced liver injury, atorvastatin, drug-induced autoimmune hepatitis

## Abstract

Drug-induced liver injury with autoimmune-like features is an uncommon yet clinically significant adverse effect of several medications, including statins. Although generally considered safe, atorvastatin has been associated with rare cases of hepatotoxicity that mimic autoimmune hepatitis on histology. We describe a 53-year-old woman with recently diagnosed chronic kidney disease who developed progressive jaundice and right upper quadrant pain shortly after starting atorvastatin. Laboratory evaluation revealed a mixed pattern of liver injury with worsening liver function tests, while serologic studies for viral hepatitis, autoimmune markers, and metabolic diseases were negative. Imaging ruled out biliary or structural abnormalities. Liver biopsy showed features compatible with autoimmune hepatitis. Given the diagnostic challenge, particularly in seronegative presentations, causality and phenotype assessment tools are essential. The updated Roussel Uclaf Causality Assessment Method (RUCAM) is a validated, widely used instrument with good reproducibility in both idiosyncratic DILI and drug-induced autoimmune hepatitis (DIAIH). Similarly, although the Simplified Autoimmune Hepatitis Score was developed for idiopathic autoimmune hepatitis, it can help identify immunemediated features in DILI when interpreted in a clinical context. In this patient, a RUCAM and S-AIH score of 6, together with compatible histology and a clear temporal association with atorvastatin, supported the diagnosis of statin-induced autoimmune-like hepatitis. The patient received corticosteroids, achieving marked clinical and biochemical improvement. This case highlights the need to consider DILI with autoimmune features in patients with unexplained liver injury after recent statin exposure. When autoimmune serologies are negative, liver biopsy and structured tools such as updated RUCAM and S-AIH are crucial to establish the diagnosis and guide timely immunosuppressive therapy. Although rare, statin-induced autoimmune-like hepatitis is serious but potentially reversible.

## Introduction

Drug-induced liver injury (DILI) is a major cause of acute liver dysfunction, presenting across a wide clinical spectrum from mild asymptomatic elevation of transaminase to acute liver failure [1]. A rare but clinically significant subtype of DILI is drug-induced autoimmune hepatitis (DIAIH), closely mimics idiopathic autoimmune hepatitis (AIH) in clinical presentation and histopathology and, in some cases, serologic profile [1]. Accurate differentiation between DIAIH and idiopathic AIH is critical, given their divergent prognostic implications and long-term management strategies. Statins, among the most widely prescribed lipid-lowering medications, are generally well tolerated; however, rare cases of immune-mediated hepatotoxicity have been reported [2, 3]. Diagnostic complexity increases in seronegative cases, where conventional autoimmune markers are absent. In such scenarios, liver biopsy and structured causality assessment tools, including the updated Roussel Uclaf Causality Assessment Method (RUCAM) and the Simplified AIH score (S-AIH), play a central role [4]. We reported a case of seronegative statin-induced DIAIH, highlighting the diagnostic value of histology and standardized scoring systems.

## Case report

**Figure 1:**
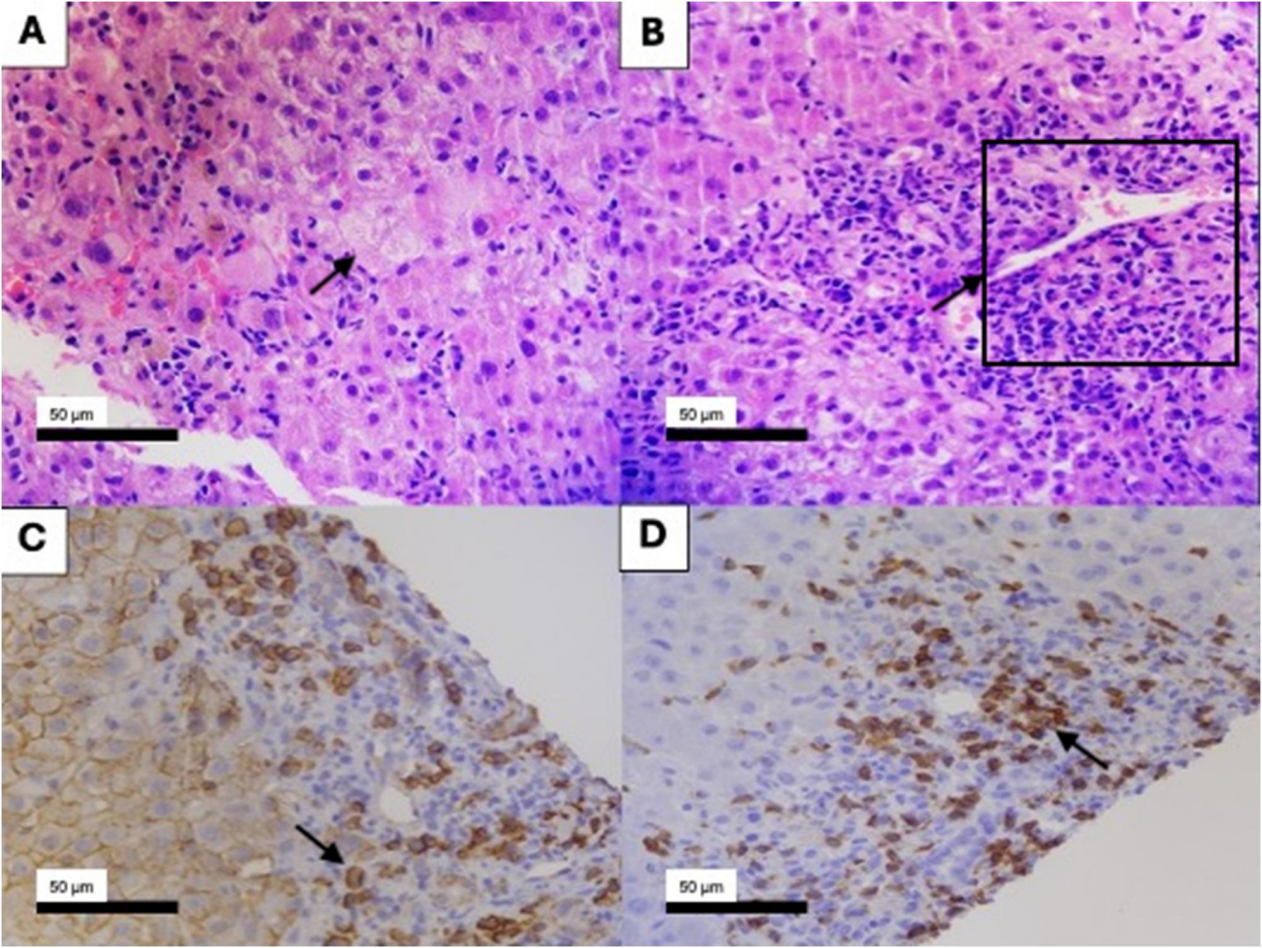
Liver biopsy. (A) Liver parenchyma with focal cholestasis present, ballooning (black arrow) (hematoxylin and eosin, magnification 40×). (B) A portal space with present, moderate inflammation, consisting of T lymphocytes, plasma cells, and B lymphocytes (black arrow) (hematoxylin and eosin, magnification 40×). (C) Highlights the plasma cells (black arrow) in the portal space in dark brown (immunohistochemistry with CD138, magnification 40×). (D) Highlighting lymphocyte infiltrate in the portal space (black arrow) (immunohistochemistry with CD3, magnification 40×).

A 53-year-old woman with hypertension and advanced chronic kidney disease (CKD KDIGO G5 Ax) was admitted with progressive jaundice, nausea, mild dyspnea, and right upper quadrant abdominal pain. Her chronic medications included dapagliflozin, finerenone, losartan, and allopurinol. Atorvastatin had been initiated 1 month earlier (January 2025). She denied alcohol consumption, paracetamol use, or herbal supplements and had no personal or family history of liver or autoimmune diseases. Physical examination revealed jaundice and diffuse abdominal tenderness. Laboratory tests demonstrated hepatocellular pattern of liver injury (R factor 7.2), with alanine aminotransferase (ALT) 938 IU/L and aspartate aminotransferase (AST) 791 IU/L. Total bilirubin was 5.3 mg/dL, with moderate elevations in alkaline phosphatase (223 IU/L) and gamma-glutamyl transferase (124 IU/L). Albumin was mildly reduced (3.2 g/dL), while globulin levels were normal (**Supplementary Table S1**). Atorvastatin was discontinued on admission (16 February 2025) because of suspected DILI, and allopurinol was withdrawn preventively. Serologic testing for hepatitis A, B, C, and E, as well as herpes simplex virus, Epstein–Barr virus, and cytomegalovirus, was negative. Ferritin and transferrin levels were normal. Abdominal ultrasound and magnetic resonance cholangiopancreatography excluded biliary obstruction or vascular abnormalities but suggested nonspecific hepatic inflammation. Autoimmune testing revealed negative antinuclear antibodies (ANA), anti-smooth muscle antibodies (ASMA), anti-liver kidney microsomal type 1 antibodies, and antimitochondrial antibodies, while immunoglobulin G was mildly elevated (1.1 × the upper limit of normal). The updated RUCAM score was 7, indicating probable DILI [Time to onset from the cessation of the drug ≤15 days (+1), decrease of ≥50% within 30 days (+2), exclusion of other causes (+2), reaction labelled in the product characteristics (+2)], and the S-AIH score was 6, consistent with probable AIH [>1.1 UL IgG (+2), Histologic findings typical of HAI (+2), negative viral markers (+2)] [4]. Liver biopsy demonstrated characteristic features of AIH, including moderate portal inflammation composed predominantly of CD3+ T lymphocytes (70%), CD138+ plasma cells (20%), and CD20+ B cells (10%), mixed lobular inflammation, focal cholestasis, microvesicular steatosis (<33%), ballooning degeneration, and regenerative changes with pseudoacinar formations and binucleation (Fig. 1). Considering the temporal relationship with statin exposure, exclusion of alternative etiologies, seronegativity, and histologic findings, a diagnosis of statin-induced DIAIH was established. Prednisone 30 mg/day was initiated on February 24, 2025. Total bilirubin peaked at 19.9 mg/dL on February 25 and subsequently declined. At discharge, bilirubin had decreased to 4.4 mg/dL, ALT to 140 IU/L, and AST to 66 IU/L. With corticosteroid tapering, the patient showed continued improvement, achieving complete biochemical recovery within 6 weeks (**Supplementary Table S1**).

## Discussion

DIAIH is an uncommon immune-mediated liver injury that may be clinically and histologically indistinguishable from idiopathic AIH and typically develops weeks to months after drug exposure [1]. Among implicated agents, statins have been reported only in isolated cases [2, 3]. Although most patients tolerate statins well, clinicians should remain aware of their potential to cause severe immune-mediated hepatotoxicity. This case highlights the diagnostic complexity of seronegative presentations. While clinical and histological findings were compatible with AIH, the absence of detectable autoantibodies complicated the diagnosis. Liver biopsy, therefore, played a decisive role and remains a cornerstone in the evaluation of suspected immune-mediated DILI [1, 5]. Importantly, a substantial proportion of idiopathic AIH cases may lack detectable autoantibodies, underscoring that negative ANA or ASMA testing does not exclude immune-mediated liver injury [1]. The mechanisms underlying statin-induced DIAIH remain incompletely understood but are thought to involve drug-induced neoantigen formation, immune activation, and host susceptibility factors [6]. Hepatic drug metabolism may generate immunogenic drug–protein adducts that trigger adaptive immune responses resembling AIH, while oxidative stress and inflammatory pathways may further contribute to immune-mediated liver injury [6]. Patients with chronic kidney disease may be particularly susceptible to idiosyncratic drug reactions due to altered drug handling and immune dysregulation, including changes in gut–liver immune interactions [7]. Clinically, statin-induced DIAIH is characterized by delayed onset, AIH-like histopathology, and a typically reversible course following statin withdrawal [2]. Although allopurinol is a recognized cause of DILI, it more commonly presents with systemic hypersensitivity features and cholestatic or granulomatous histologic patterns, which were absent in this case [1, 5]. Structured causality assessment is therefore essential. The updated RUCAM score is a validated and reproducible tool for evaluating idiosyncratic DILI and DIAIH, while the S-AIH score may assist in identifying immune-mediated features when interpreted in the appropriate clinical context [4, 8–9]. In our patient, these tools reflected the characteristic overlap observed in DIAIH. Management consists primarily of drug withdrawal and corticosteroid therapy, leading to clinical and biochemical improvement in most reported cases [2, 10]. In this patient, the rapid response to prednisone further supported an immune-mediated mechanism. Despite underlying CKD, the benefits of timely immunosuppression outweighed potential risks, resulting in complete recovery without relapse.

## Conclusions

This case highlights the importance of considering DIAIH in patients with unexplained hepatocellular injury, even in the absence of autoimmune serologic markers. Although statins are rarely implicated, they can cause clinically significant immune-mediated hepatotoxicity. In seronegative or diagnostically equivocal cases, liver biopsy and structured causality assessment are essential. Early recognition and appropriate immunosuppressive therapy may result in full recovery and prevent progression to acute liver failure.

## Supplementary Material

goag022_Supplementary_Data
