# Supplementary material for: Drug-induced autoimmune hepatitis due to atorvastatin: a complex clinical case and literature review
Source: Gastroenterol Rep (Oxf). 2026 Mar 7;14:goag022. doi: 10.1093/gastro/goag022 (PMC12967216; doi:10.1093/gastro/goag022)
Supplement: goag022_Supplementary_Data [file goag022_supplementary_data.docx]

**Supplementary Table 1**. Lab tests evolution

|  | **Admission(16.02.2025)** | 19.02.2025 | **20.02.2025**  **(Liver biopsy)** | 24.02.2025 | **PDN start dose (25.02.2025)** | (28.02.2025) | (03.03.2025) | (07.03.2025) | **Discharge**  **(10.03.2025)** |
| --- | --- | --- | --- | --- | --- | --- | --- | --- | --- |
| **TB**  **mg/dL** | 5.3 | 12.2 | 14.3 | 19.0 | 19.9 | 14.8 | 10.6 | 5.9 | 4.4 |
| **DB**  **mg/dL** | 3.6 | 8.3 | 9.4 | 15.0 | 15.3 | 8.7 | 6 | 3.1 | 2.2 |
| **IB**  **mg/dL** | 1.7 | 3.8 | 4.96 | 4.03 | 4.60 | 6.1 | 4.59 | 2.8 | 2.2 |
| **Alb**  **g/dL** | 3.2 | 2.8 | 2.63 | 2.44 | 2.54 | 2.21 | 2.42 | 2.5 | 2.5 |
| **Glob**  **g/dL** | 3.2 | 3.1 | 3.1 | 2.7 | 2.9 | 2.2 | 2.6 | 2.3 | 2.2 |
| **ALT**  **IU/L** | 938 | 770 | 675 | 337 | 369 | 368 | 307 | 191 | 140 |
| **AST**  **IU/L** | 791 | 643 | 560 | 559 | 691 | 505 | 262 | 97 | 66 |
| **GGT**  **IU/L** | 216 | 142 | 136 | 160 | 189 | 576 | 572 | 433 | 346 |
| **ALP**  **IU/L** | 394 | 288 | 282 | 264 | 268 | 571 | 636 | 425 | 356 |
| **LDH**  **IU/L** | 336 | 374 | 345 | 1107 | 1634 | 1087 | 835 | 487 | 346 |
| **IgG**  **mg/dL** |  |  | 1753 |  |  |  | 1184 |  |  |

PDN: prednisone, TB: total bilirubin, DB: direct bilirubin, IB: indirect bilirubin, ALB: albumin, Glob: globulin. ALT: alanine aminotransferase, AST: aspartate aminotransferase, GGT: gamma-glutamyl transferase, ALP: alkaline phosphatase, LDH: lactate dehydrogenase, IgG: immunoglobulin G.
